# High Density of NRF2 Expression in Malignant Cells Is Associated with Increased Risk of CNS Metastasis in Early-Stage NSCLC

**DOI:** 10.3390/cancers13133151

**Published:** 2021-06-24

**Authors:** Georgios Tsakonas, Alfonso Martín-Bernabé, Konstantinos Rounis, Pablo Moreno-Ruiz, Johan Botling, Luigi De Petris, Antti Ylipää, Artur Mezheyeuski, Patrick Micke, Arne Östman, Simon Ekman

**Affiliations:** 1 Thoracic Oncology Center, Theme Cancer, Karolinska University Hospital, Solna, 17164 Stockholm, Sweden; kostas@rounis.gr (K.R.); luigi.depetris@ki.se (L.D.P.); simon.ekman@ki.se (S.E.); 2 Department of Oncology–Pathology, Karolinska Institutet, 17164 Stockholm, Sweden; alfonso.martin.bernabe@ki.se (A.M.-B.); pablo.moreno.ruiz@ki.se (P.M.-R.); arne.ostman@ki.se (A.Ö.); 3 Department of Immunology, Genetics and Pathology, Uppsala University, 75185 Uppsala, Sweden; johan.botling@igp.uu.se (J.B.); artur.mezheyeuski@igp.uu.se (A.M.); patrick.micke@igp.uu.se (P.M.); 4 Genevia Technologies Oy, 33100 Tampere, Finland; antti.ylipaa@geneviatechnologies.com

**Keywords:** brain metastases, non-small cell lung cancer, NRF2, TrxR1

## Abstract

**Simple Summary:**

We retrospectively analyzed 304 patients with surgically removed non-small cell lung cancer (NSCLC). Multiplex antibody staining of nuclear factor erythroid 2-related factor 2 (NRF2) and thioredoxin reductase 1 (TrxR1) was conducted and scored in cytokeratin-positive (CK+) cells within the whole-tissue core as well as the tumor and stromal compartments of each tissue microarray (TMA) core. A high density of NRF2+/CK+ cells in the whole-tissue core compartment was an independent prognostic factor, with an eightfold increase in odds regarding the risk of relapse in the central nervous system (CNS). This is the first study to report a tumor-cell-associated protein biomarker for CNS relapse in early-stage lung cancer and the first trial to report the correlation between NRF2 expression and risk of CNS relapse. The results of our study may have an impact on the follow-up strategy for early-stage NSCLC patients and eventually improve their prognosis.

**Abstract:**

Nuclear factor erythroid 2-related factor 2 (NRF2) protein expression promotes cancer progression in non-small cell lung cancer (NSCLC). However, its role in the clinical setting has not been established. We retrospectively analyzed data from 304 patients with surgically removed NSCLC. Multiplex antibody staining of NRF2 and thioredoxin reductase 1 (TrxR1) was conducted and scored in cytokeratin-positive (CK+) cells within the whole-tissue core as well as the tumor and stromal compartments of each tissue microarray (TMA) core. A high density of NRF2+/CK+ cells in the whole-tissue core compartment was correlated with a higher risk of central nervous system (CNS) relapse OR = 7.36 (95% CI: 1.64–33.06). The multivariate analysis showed an OR = 8.00 (95% CI: 1.70–37.60) for CNS relapse in NRF2+/CK+ high-density cases. The density of TrxR1+/CK+ cells failed to show any statistically significant risk of relapse. The OS analyses for NRF2+/CK+ and TrxR1+/CK+ cell density failed to show any statistical significance. This is the first study to report a correlation between NRF2+/CK+ cell density and the risk of CNS relapse in early-stage NSCLC. The results of our study may impact the follow-up strategy for early-stage NSCLC patients and eventually improve their prognosis.

## 1. Introduction

Lung cancer is the leading cause of cancer-related death among all solid tumor cases [1]. The dissemination of NSCLC in the central nervous system (CNS) is an adverse prognostic factor and is associated with an increased incidence in adenocarcinoma and tumors harboring oncogenic driver mutations [2,3]. Prognosis is significantly better in patients with oncogenic drivers, where high response rates and prolonged survival can be achieved with the use of targeted therapies, although this applies to a minority of NSCLC patients [4,5,6,7,8,9,10,11,12,13,14,15,16]. 

The transcription factor nuclear factor erythroid 2-related factor 2 (NRF2) is a vital component of the cellular antioxidant response, and its activation in cancer cells promotes cancer progression and metastasis [17,18,19]. NRF2 is involved in the expression of genes that affect cell proliferation, such as *NOTCH1, NPNT, BMPR1A, IFG1, ITGB2, PDGFC, VEGFC* and *JAG1* [20,21]. In pancreatic cancer cells, NRF2 supports cell proliferation and metabolism through the regulation of cap-dependent and cap-independent mRNA translation [22]. A key study by DeNicola et al. showed that NRF2 promoted K-ras^G12D^-initiated pancreatic and lung tumorigenesis as well as proliferation in cancer cell lines and human pancreatic cancer tissue. Furthermore, K-ras^G12D^, BRAF^V619E^ and c-Myc^ERT2^ oncogenic signaling was related to increased mRNA and protein levels of NRF2 and its target genes [23]. Another study by Aljohani et al. showed that mutations in the *Keap1*–*Nrf2* anti-oxidant response elements (ARE) pathway enhance the circulating tumor cells’ (CTCs) ability to metastasize in distant organs. These mutations were found in the majority of NSCLC patients that metastasized to the brain [24]. Thioredoxin reductase 1 (TrxR1) is the cytosolic isoenzyme of the three different TrxRs found in human cells. Its role in cancer biology is less clear since it may protect normal cells from carcinogenesis but may also promote cancer progression [25]. There is a correlation between NRF2 and TrxR1 expression; activated NRF2, as a result of cellular oxidative stress, forms a complex with the antioxidant/electrophile responsive element (ARE/EpRE) in the promoter region in a plethora of genes, including Trx1, activating their expression [26]. In addition, a potential mechanism of NRF2 activation through the depletion of cytosolic Trx1 levels has been demonstrated in preclinical models [27]. 

The aim of our study was to investigate whether a high expression of NRF2 or TrxR1 in cytokeratin-positive (CK+) cells, i.e., cancer cells, in early-stage NSCLC is predictive of relapse in the CNS or other organs. We focused mainly on the risk of CNS relapse due to the dismal prognosis of this patient category. 

## 2. Results

### 2.1. Patient Characteristics

**Figure 1:**
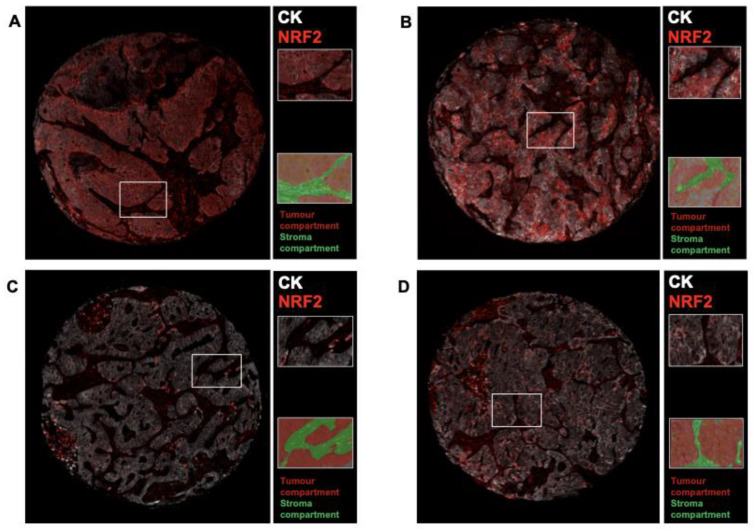
Representative multiplex immunofluorescence images from whole-tissue core biopsies of two patients with lung adenocarcinoma with a high (**A**,**B**) and low (**C**,**D**) density of cells stained positive for both CK (white) and NRF2 (red). Each tissue core includes zoomed-in images of the corresponding regions marked in the solid white box and a tissue segmentation map of tumor epithelial (red) and stroma (green) areas. Images were taken at 20× magnification (0.5 µm/pixel).

The demographics of the entire cohort are presented in Table 1. We had information about quantified NRF2+ cell and TrxR1+ cell density in the different tissue compartments for all 304 patients (Figure 1). The mean age of the patients in our cohort was 67.5 (±7.6) years and 50% were male. A total of 258 patients were included in the final analysis due to missing data regarding tumor relapse. Among these patients, 56.6% did not experience a relapse, while the most common relapse site was the thoracic cavity (22.9%). Sixteen patients had a relapse in the CNS (6.2%), of which 14 (87.5%) had a high cell density of NRF2+/CK+ (Table 2). All of these patients presented with symptomatic brain metastatic disease, and the CNS was the only metastatic site at the time of relapse.

### 2.2. Survival Analysis

**Figure 2:**
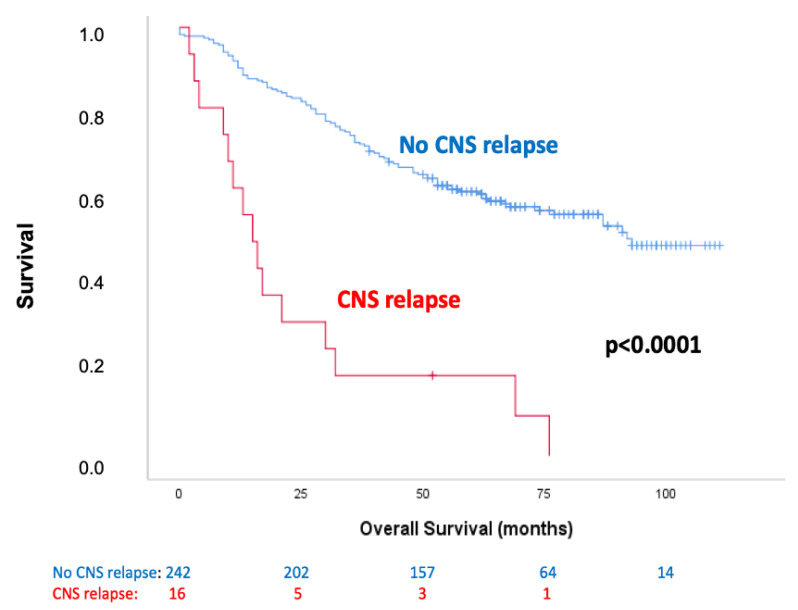
Kaplan–Meier curve of OS in patients who relapsed in the CNS compared to that in the remaining patients.

The OS analyses of the cell density of NRF2+/CK+ and TrxR1+/CK+ cells in the different tissue compartments failed to show any statistical significance. Patients that relapsed in the CNS had a significantly worse OS (*p* < 0.0001) when compared to that of the remaining patients (patients that did not relapse or relapsed in sites other than the CNS) (Figure 2).

### 2.3. Risk of Relapse: Univariate Analyses and Chi-Square Test

The density of TrxR1+/CK+ cells in the different tissue compartments failed to show any statistically significant risk of relapse, relapse only in the CNS or relapse in other sites. 

The same analyses were conducted for NRF2+/CK+ cell density, where a significantly higher risk of relapse in the CNS (patients with CNS relapse vs. those without it) was observed in the group with high NRF2+/CK+ cell density in the whole-tissue core, with an OR of 7.36 (95% CI: 1.64–33.06) and a chi-square *p*-value of 0.003 (Table 3). The OR for relapse in the CNS was 3.08 (95% CI: 0.97–9.81) for NRF2+/CK+ high cell density in the tumor compartment and 3.05 (95% CI: 0.96–9.72) in the stroma compartment. Chi-square *p*-values for the abovementioned compartments were 0.068 and 0.069, respectively. In general, there was no difference observed in the risk of relapse in the different tissue compartments. 

Age, sex, stage and histology (adenocarcinoma vs. non-adenocarcinoma) were not found to be significant predictors of risk of relapse in the CNS (Table 3). The epithelial density, defined as CK positivity in the total tissue core or in the different compartments, did not affect the risk of relapse in general or relapse in the CNS or other sites.

### 2.4. Multivariate CNS Metastasis Risk Analysis in the Whole-Tissue Core Compartment

Despite not being significant in the univariate analysis, age, histology and stage were deemed to be clinically significant variables and were included in the multivariate analysis, together with CK+/NRF2+ cell density. High cell density of CK+/NRF2+ in the whole-tissue core was an independent positive predictive factor for CNS relapse, whereas no other variables were statistically significant (Table 3).

The optimal cut-off value for NRF2+/CK+ cell density was then further explored. The distribution of NRF2+/CK+ cell density in the whole-tissue core compartment was not normally distributed, with a skewness of 4.6 and kurtosis of 28.7 (Figure S1). The ROC analysis failed to show another optimal cut-off value, and the median NRF2+/CK+ cell density was determined to be the most optimal cut-off for our cohort (Figure S2).

## 3. Discussion

A high cell density of CK+/NRF2+ in the whole-tissue core compartment was an independent prognostic factor regarding the risk of relapse in the CNS in our cohort of early-stage lung cancer patients. To our knowledge, this is the first study to report a predictive tumor-cell-associated protein biomarker for CNS relapse in early-stage lung cancer.

The role of TrxR1 in tumorigenesis and cancer progression is not fully understood, and no significant findings related to the cell density of TrxR1+/CK+ in the different TMA compartments were observed in our study [25]. NRF2 expression, on the other hand, seems to play a more important role in cancer progression and metastasis. This role is most probably mediated through the promotion of expression of certain genes that are vital for cell proliferation and metabolism [20,21,22]. A recently published trial from Taiwan showed that cytoplasmic NRF2 expression in early-stage NSCLC was correlated with a worse prognosis and response to cisplatin-based chemotherapy after relapse [28]. In another trial conducted with early-stage NSCLC patients, cytoplasmic NRF2 expression, as well as expression of its stabilizing protein DJ1, was independent prognostic factors for poorer OS [29]. In our study, we did not evaluate the cytoplasmic expression of NRF2, although a high cell density of CK+/NRF2+ in the different tissue compartments did not influence OS. An eightfold increase in odds of a CNS relapse was observed in patients whose whole tumor compartments stained positive for NRF2 in our study. The external validation of this finding is important and will be a focus of future studies.

The early detection of CNS relapse may prove to be of vital importance for this patient category. The profound difference in OS between patients that relapsed in the CNS versus the remaining patients in our cohort highlights the need to find a better follow-up and treatment strategy for these patients. The earlier detection of CNS relapse in asymptomatic patients can be associated with a considerably greater PS, a lesser tumor burden in the CNS and the absence of extracranial disease and it could potentially lead to increased overall survival for these individuals. This assumption is derived from evidence regarding the prognosis of these patients, where surgery for a single metastasis, better PS, a lower number of CNS metastases and the absence of extracranial disease are associated with a longer OS [30,31]. The follow-up strategy for patients that have a higher risk of CNS relapse after surgery should include brain MRIs in regular intervals. 

The major limitation of our study is the retrospective nature making it prone to selection bias. A certain risk of information bias regarding missing follow-up data about tumor relapse also exists. Information bias regarding IHC scoring cannot be avoided, although it is considered limited in our cohort due to the utilized methodology. Overfixation or underfixation during IHC staining can also cause false positive or false negative results, respectively. Another limitation in all similar published studies is intratumoral heterogeneity, which may render TMAs as not being representative of the entire tumor. On the other hand, the overfitting of data was avoided by choosing the median IHC expression value as a cut-off in order to define high vs. low protein expression. Further statistical analyses with ROC curves did not reveal any other optimal cut-off value. This methodology renders the protein expression analysis as unbiased. The size of our cohort was large enough in order to perform the planned statistical analyses, even though the number of patients with CNS relapse was relatively small, something which is expected in a cohort of early-stage NSCLC patients. The real-world nature of our study strengthens the external validity of our results.

## 4. Materials and Methods

### 4.1. Patient Population

We designed a retrospective cohort study consisting of 304 patients with surgically removed NSCLC. We collected information on the physician’s evaluation of performance status (PS) at the time of diagnosis, age at diagnosis, histology, site of first relapse after primary tumor surgery, pathological stage of disease (TNM version 7), smoking status and gender (Table 1). The tissue microarray (TMA) cohort was based on diagnostic tissue from NSCLC patients who received surgical treatment from 1/1/2006 to 30/12/2010 at the Academic University Hospital in Uppsala, Sweden, and histopathological data for parts of this cohort have been reported previously [32,33].

### 4.2. Multiplex Immunofluorescence Staining and Scanning

Multiplexed immunofluorescence staining was conducted on tissue cores mounted on TMAs from surgically removed lung tumors. The TMA was constructed using a manual tissue arrayer (MTA-1, Beecher Instruments, Sun Prairie, CA, USA), as previously described [34,35]. All tumors were included in duplicates (2 × 1 mm tissue cores). Four-micrometer sections were cut from the formalin-fixed, paraffin-embedded (FFPE) tissue blocks mounted on adhesive slides and baked at 60 °C for 45 min. TMA blocks were successfully constructed for all patients. 

The Opal multiplexed stained method was performed as previously described [36]. Briefly, TMA slides cut from FFPE blocks were deparaffinized and rehydrated with a serial passage through changes in xylene, graded ethanol and distilled water. Multiplexed IHC staining was performed using the Opal technology (Akoya Biosciences, Menlo Park, CA, USA) for the simultaneous detection of NRF2 (ab62352; 1:500; Abcam, Cambridge, UK) and TrxR1 (B-2 clone, sc-28321; 1:100; Santa Cruz Biotechnology, Santa Cruz, CA, USA). The detection of cytokeratin and epithelial tissue was achieved by the combination of pan-cytokeratin (AE1/AE3 clone; 1:500; Dako, Glostrup, Denmark), pan-cytokeratin (C-11 clone, ab7753; 1:100; Abcam, Cambridge, UK) and E-cadherin (36 clone, 610182; 1:4000; BD Biosciences, San Jose, CA, USA). Cells that were positive for this antibody cocktail are hereafter referred to as CK+ cells.

ImmPRESS HRP Anti-Rabbit /-Mouse IgG Peroxidase Polymer Detection Kits (Vector Laboratories, Burlingame, CA, USA) were used as secondary antibodies. Tyramide signal amplification (TSA) Opal-conjugated detection reagents (Opal 520, Opal 570 and Opal 690) were used to increase the fluorescent signal while allowing microwave treatment to remove primary and secondary antibodies. Nuclei were stained with DAPI and mounted with a Prolong Diamond Antifade Mountant (Thermo Scientific, Waltham, MA, USA). Images were acquired by the Vectra Polaris instrument (Akoya Biosciences, Menlo Park, CA, USA) as described previously [36].

### 4.3. Image Analysis: Cell Segmentation and Fluorescence Intensity Measurements

For quantitative analysis, multiplexed images were initially analyzed using inForm software (2.4.8 version, Akoya Biosciences, Menlo Park, CA, USA). Cell segmentation was carried out using the DAPI-stained nuclei signal. InForm software was trained to identify three compartments or areas: tumor, stroma and excluded (blank) areas. Tumor epithelial areas were defined as epithelial cells that expressed cytokeratin (CK+), whereas areas not expressing cytokeratin (CK-) were identified as stroma. Necrotic areas as well as staining artefacts were manually excluded from analysis.

We extracted the mean normalized counts for each marker from the output files created by the imaging system as well as the area of each tissue category in each multiplexed image. To classify the extracted intensity values into marker-positive and marker-negative categories, we modeled the data for each marker over all samples with a mixed chi-squared distribution and a normal distribution. We assumed that the intensity values corresponding to marker-positive samples were normally distributed, and chi-squared distribution was used to model the noise component of marker-negative samples. The threshold value for each marker was determined to be at the intersection of the two density functions. 

For each patient, we computed CK density values by dividing CK+ cells by the whole-tissue core (total area) and separately by computing the area classified as tumor and stroma. We also computed the density of NRF2 and CK double-positive cells in the whole-tissue core, tumor and stroma. The same metrics were computed for TrxR1 following NRF2 analysis.

### 4.4. Statistical Analysis

Descriptive statistics were performed in order to analyze demographic and clinical characteristics. Overall survival, defined as the time from diagnosis to the date of death by any cause or last date of follow-up (censoring date was 10 October 2015), was assessed using the Kaplan–Meier method. The log-rank test was used to calculate differences in OS between different subgroups.

The median value of every protein IHC expression was initially tested as a cut-off to define high versus low expression. This was carried out in order to have an unbiased first analysis. The protein expressions, which were found to be significant in the first analysis, were thereafter tested for an optimal cut-off with ROC curves. The distribution of the proteins, which was statistically significant, was tested with skewness and kurtosis. The optimal cut-off value was decided after all of these analyses were conducted.

The primary outcome was relative risk of relapse in the CNS or other organ after surgery, and it was calculated by binary logistic regression. Odds ratios (OR) with 95% confidence intervals (CI) were calculated using univariate and multivariate regression analysis. Univariate logistic regression analysis was undertaken with the following independent variables: NRF2+/CK+ cell density in the three different tissue compartments (whole-tissue core, tumor and stroma), TrxR1+/CK+ cell density in the same three different tissue compartments, performance status (PS), age at diagnosis, histology (adenocarcinoma vs. non-adenocarcinoma), pathological stage of the disease and gender. The variables that were found to be significant in predicting the risk of relapse, as well as the variables that were deemed to be clinically significant predictors of the risk of relapse, were included in the multivariate regression models. Fisher’s exact test was also implemented in order to test the correlation between tumor relapse and marker status. 

All tests were two-sided, and statistical significance was considered at a 5% level. Statistical analyses were performed using the Statistical Package for Social Sciences (SPSS) version 25.

## 5. Conclusions

This is, to our knowledge, the first study to report a predictive tumor-cell-associated protein biomarker for CNS relapse in early-stage NSCLC and the first study to report the correlation between NRF2 expression in CK+ cells and the risk of CNS relapse. The external validation of these results is required prior to their clinical implication. The results of our trial may alter the follow-up strategy of early-stage NSCLC patients and eventually improve OS for NRF2-positive cases.

## Figures and Tables

**Table 1:** Baseline demographics and disease characteristics. In square brackets, the absolute number of patients in each subgroup and the total number of patients with available information are shown.

Variable	Frequency
Gender (% male)	50 [152/304]
Age (mean ± SD)	67.5 ± 7.6
*Histology (%)* Adenocarcinoma Squamous cell Large cell Adenosquamous NOS/mixed type	57.6 [175/304] 29.9 [91/304] 8.2 [25/304] 3 [9/304] 1.4 [4/304]
*Stage at diagnosis* IA IB IIA IIB IIIA IIIB	38.8 [118/304] 22.7 [69/304] 10.5 [32/304] 10.2 [31/304] 14.5 [44/304] 3.3 [10/304]
*Smoking status (%)* Current Ex-smoker (>1 year) Never smoked	51.6 [157/304] 36.5 [111/304] 11.8 [36/304]
*Relapse site (%)* No relapse CNS Thoracic cavity Bone Liver Adrenal Multiple organs	56.6 [146/258] 6.2 [16/258] 22.9 [59/258] 4.6 [12/258] 1.9 [5/258] 0.4 [1/258] 7.4 [19/258]
NRF2 in cancer cells, + vs. − (%)	50 [152/304]

NOS: not otherwise specified, SD: standard deviation, NRF2: transcription factor nuclear factor erythroid 2-related factor 2, CNS: central nervous system.

**Table 2:** Cross-tabs: correlation between NRF2+/CK+ cell density and risk of CNS relapse.

Variable		Non-CNS Relapse/Relapse Free	CNS Relapse	Total Row
NRF2+/CK+ cell density	Low	124	2	126
	High	118	14	132
Total column		242	16	258

NRF2: transcription factor nuclear factor erythroid 2-related factor 2, CNS: central nervous system, CK: cytokeratin.

**Table 3:** Binary logistic regression analysis for the patients who relapsed in the CNS vs. those who did not.

Univariate Analysis	Odds Ratio (95%CI)	*p*–Value
NRF2 (+ vs. −)	7.36 (1.64–33.06)	0.009
Sex (female vs. male)	0.79 (0.29–2.19)	0.652
Age *	0.96 (0.90–1.02)	0.198
Histology (adenocarcinoma vs. rest)	1.02 (0.37–2.81)	0.997
*Stage* IA IB IIA IIB IIIA IIIB	Ref 2.56 (0.69–9.45) 2.15 (0.37–12.47) 1.08 (0.12–10.09) 2.18 (0.47–10.26) –	Ref 0.158 0.393 0.949 0.322 0.999
**Multivariate analysis**		
NRF2 (+ vs. −)	8.00 (1.70–37.60)	0.009
Age *	0.95 (0.88–1.01)	0.106
Histology (adenocarcinoma vs. rest)	1.08 (0.36–3.26)	0.891
*Stage* IA IB IIA IIB IIIA IIIB	Ref 2.43 (0.60–9.84) 3.19 (0.47–21.59) 0.92 (0.09–9.12) 1.36 (0.28–6.68) –	Ref 0.212 0.234 0.941 0.706 0.999

CI: confidence interval, Ref: reference, * age was analyzed as a continuous variable.

## Data Availability

The data presented in this study are available on request from the corresponding author. The data are not publicly available due to ethical restrictions from the institutional review boards of Akademiska University Hospital, Uppsala, Sweden (diary numbers: 2006/325 and 2012/532) and Uppsalas Biobank (BbA–827-2018-058).

## Supplementary Materials

The following are available online at https://www.mdpi.com/article/10.3390/cancers13133151/s1, Figure S1: Distribution of NRF2+/CK+ cell density in the whole-tissue core compartment. Figure S2: Receiver operating characteristic (ROC) curve demonstrating the sensitivity and specificity of the median NRF2+/CK+ cell density in the whole-tissue compartment as a cut-off value on the probability of developing brain metastases.

## References

[1] Fitzmaurice C., Dicker D., Pain A., Hamavid H., Moradi-Lakeh M., MacIntyre M.F., Allen C., Hansen G., Woodbrook R., Global Burden of Disease Cancer Collaboration (2015). The global burden of cancer 2013. JAMA Oncol..

[2] Langer C.J., Mehta M.P. (2005). Current management of brain metastases, with a focus on systemic options. J. Clin. Oncol..

[3] Ferlay J., Colombet M., Soerjomataram I., Mathers C., Parkin D.M., Pineros M., Znaor A., Bray F. (2019). Estimating the global cancer incidence and mortality in 2018: GLOBOCAN sources and methods. Int. J. Cancer.

[4] Gadgeel S.M., Shaw A.T., Govindan R., Gandhi L., Socinski M.A., Camidge D.R., Ou S.H.I. (2016). Pooled Analysis of CNS Response to Alectinib in Two Studies of Pretreated Patients with ALK-Positive Non–Small-Cell Lung Cancer. J. Clin. Oncol..

[5] Solomon B.J., Cappuzzo F., Felip E., Blackhall F.H., Costa D.B., Kim D.W., Nakagawa K., Wu Y.L., Mekhail T., Paolini J. (2016). Intracranial efficacy of crizotinib versus chemotherapy in patients with advanced ALK-positive non-small-cell lung cancer: Results from PROFILE 1014. J. Clin. Oncol. Off. J. Am. Soc. Clin. Oncol..

[6] Crino L., Ahn M.J., De Marinis F., Groen H.J., Wakelee H., Hida T., Mok T., Spigel D., Felip E., Nishio M. (2016). Multicenter phase II study of whole-body and intracranial activity with ceritinib in patients with ALK-rearranged non-small-cell lung cancer previously treated with chemotherapy and crizotinib: Results from ASCEND-2. J. Clin. Oncol.

[7] Felip E., Crino L., Kim D.W., Spigel D.R., Nishio M., Mok T., Scagliotti G., Cesic D., Sutradhar S., Shaw A.T. (2016). 141PD: Whole body and intracranial efficacy of ceritinib in patients (pts) with crizotinib (CRZ) pretreated, ALK-rearranged (ALK+) non-small cell lung cancer (NSCLC) and baseline brain metastases (BM): Results from ASCEND-1 and ASCEND-2 trials. J. Thorac. Oncol. Off. Publ. Int. Assoc. Study Lung Cancer.

[8] Felip E., Orlov S., Park K.-S. (2015). ASCEND-3: A single-arm, open-label, multicenter phase II study of ceritinib in ALKi-naïve adult patients (pts) with ALK-rearranged (ALK+) non-small cell lung cancer (NSCLC). J. Clin. Oncol..

[9] Rosell R., Gettinger S.N., Bazhenova L.A., Langer C.J., Salgia R., Shaw A.T., Narasimhan N.I., Dorer D.J., Kerstein D., Camidge D.R. (2016). 1330: Brigatinib efficacy and safety in patients (Pts) with anaplastic lymphoma kinase (ALK)-positive (ALK+) non-small cell lung cancer (NSCLC) in a phase 1/2 trial. J. Thorac. Oncol. Off. Publ. Int. Assoc. Study Lung Cancer.

[10] Solomon B.J., Bauer T.M., Felip E., Besse B., James L.P., Clancy J.S., Klamerus K.J., Martini J.-F., Abbattista A., Shaw A.T. (2016). Safety and efficacy of lorlatinib (PF-06463922) from the dose-escalation component of a study in patients with advanced ALK+ or ROS1+ non-small cell lung cancer (NSCLC). J. Clin. Oncol.

[11] Wu C., Li Y.L., Wang Z.M., Li Z., Zhang T.X., Wei Z. (2007). Gefitinib as palliative therapy for lung adenocarcinoma metastatic to the brain. Lung Cancer.

[12] Wu Y.L., Yang J.-J., Zhou C., Feng J., Lu S., Song Y., Huang C., Wu G., Cheng Y., Zhang L. (2017). PL03.05: BRAIN: A phase III trial comparing WBI and chemotherapy with icotinib in NSCLC with brain metastases harboring EGFR mutations (CTONG 1201). J. Thorac. Oncol..

[13] Welsh J.W., Komaki R., Amini A., Munsell M.F., Unger W., Allen P.K., Chang J.Y., Wefel J.S., McGovern S.L., Garland L.L. (2013). Phase II trial of erlotinib plus concurrent whole-brain radiation therapy for patients with brain metastases from non-small-cell lung cancer. J. Clin. Oncol. Off. J. Am. Soc. Clin. Oncol..

[14] Kim J.E., Lee D.H., Choi Y., Yoon D.H., Kim S.W., Suh C., Lee J.S. (2009). Epidermal growth factor receptor tyrosine kinase inhibitors as a first-line therapy for never-smokers with adenocarcinoma of the lung having asymptomatic synchronous brain metastasis. Lung Cancer.

[15] Sperduto P.W., Wang M., Robins H.I., Schell M.C., Werner-Wasik M., Komaki R., Souhami L., Buyyounouski M.K., Khuntia D., Demas W. (2013). A phase 3 trial of whole brain radiation therapy and stereotactic radiosurgery alone versus WBRT and SRS with temozolomide or erlotinib for non-small cell lung cancer and 1 to 3 brain metastases: Radiation Therapy Oncology Group 0320. Int. J. Radiat. Oncol. Biol. Phys..

[16] Katayama T., Shimizu J., Suda K., Onozato R., Fukui T., Ito S., Hatooka S., Sueda T., Hida T., Yatabe Y. (2009). Efficacy of erlotinib for brain and leptomeningeal metastases in patients with lung adenocarcinoma who showed initial good response to gefitinib. J. Thorac. Oncol. Off. Publ. Int. Assoc. Study Lung Cancer.

[17] Wang H., Liu X., Long M., Huang Y., Zhang L., Zhang R., Zheng Y., Liao X., Wang Y., Liao Q. (2016). NRF2 activation by antioxidant antidiabetic agents accelerates tumor metastasis. Sci. Transl. Med..

[18] Satoh H., Moriguchi T., Takai J., Ebina M., Yamamoto M. (2013). Nrf2 prevents initiation but accelerates progression through the Kras signaling pathway during lung carcinogenesis. Cancer Res..

[19] Tao S., Rojo de la Vega M., Chapman E., Ooi A., Zhang D.D. (2018). The effects of NRF2 modulation on the initiation and progression of chemically and genetically induced lung cancer. Mol. Carcinog..

[20] Wakabayashi N., Shin S., Slocum S.L., Agoston E.S., Wakabayashi J., Kwak M.K., Misra V., Biswal S., Yamamoto M., Kensler T.W. (2010). Regulation of notch1 signaling by nrf2: Implications for tissue regeneration. Sci. Signal..

[21] Malhotra D., Portales-Casamar E., Singh A., Srivastava S., Arenillas D., Happel C., Shyr C., Wakabayashi N., Kensler T.W., Wasserman W.W. (2010). Global mapping of binding sites for Nrf2 identifies novel targets in cell survival response through ChIP-Seq profiling and network analysis. Nucleic Acids Res..

[22] Chio I.I.C., Jafarnejad S.M., Ponz-Sarvise M., Park Y., Rivera K., Palm W., Wilson J., Sangar V., Hao Y., Ohlund D. (2016). NRF2 promotes tumor maintenance by modulating mRNA translation in pancreatic cancer. Cell.

[23] DeNicola G.M., Karreth F.A., Humpton T.J., Gopinathan A., Wei C., Frese K., Mangal D., Yu K.H., Yeo C.J., Calhoun E.S. (2011). Oncogene-induced Nrf2 transcription promotes ROS detoxification and tumorigenesis. Nature.

[24] Aljohani H.M., Aittaleb M., Furgason J.M., Amaya P., Deeb A., Chalmers J.J., Bahassi E.M. (2018). Genetic mutations associated with lung cancer metastasis to the brain. Mutagenesis.

[25] Arner E.S.J. (2017). Targeting the selenoprotein thioredoxin reductase 1 for anticancer therapy. Adv. Cancer Res..

[26] Brigelius-Flohé R., Flohé L. (2011). basic principles and emerging concepts in the redox control of transcription factors. Antioxid. Redox Signal..

[27] Patterson A.D., Carlson B.A., Li F., Bonzo J.A., Yoo M.-H., Krausz K.W., Conrad M., Chen C., Gonzalez F.J., Hatfield D.L. (2013). Disruption of thioredoxin reductase 1 protects mice from acute acetaminophen-induced hepatotoxicity through enhanced NRF2 activity. Chem. Res. Toxicol..

[28] Chen M.J., Lin P.L., Wang L., Cheng Y.M., Chen C.Y., Lee H. (2020). Cytoplasmic, but not nuclear Nrf2 expression, is associated with inferior survival and relapse rate and response to platinum-based chemotherapy in non-small cell lung cancer. Thorac. Cancer.

[29] Merikallio H., Paakko P., Kinnula V.L., Harju T., Soini Y. (2012). Nuclear factor erythroid-derived 2-like 2 (Nrf2) and DJ1 are prognostic factors in lung cancer. Hum. Pathol..

[30] Sperduto P.W., Chao S.T., Sneed P.K., Luo X., Suh J., Roberge D., Bhatt A., Jensen A.W., Brown P.D., Shih H. (2010). Diagnosis-specific prognostic factors, indexes, and treatment outcomes for patients with newly diagnosed brain metastases: A multi-institutional analysis of 4259 patients. Int. J. Radiat. Oncol. Biol. Phys..

[31] Tsakonas G., De Petris L., Ekman S. (2017). Management of brain metastasized non-small cell lung cancer (NSCLC)—From local treatment to new systemic therapies. Cancer Treat. Rev..

[32] Botling J., Edlund K., Lohr M., Hellwig B., Holmberg L., Lambe M., Berglund A., Ekman S., Bergqvist M., Ponten F. (2013). Biomarker discovery in non-small cell lung cancer: Integrating gene expression profiling, meta-analysis, and tissue microarray validation. Clin. Cancer Res..

[33] Tsakonas G., Botling J., Micke P., Rivard C., LaFleur L., Mattsson J., Boyle T., Hirsch F.R., Ekman S. (2019). c-MET as a biomarker in patients with surgically resected non-small cell lung cancer. Lung Cancer.

[34] Nocito A., Kononen J., Kallioniemi O.P., Sauter G. (2001). Tissue microarrays (TMAs) for high-throughput molecular pathology research. Int. J. Cancer.

[35] Segersten M.U., Edlund E.K., Micke P., de la Torre M., Hamberg H., Edvinsson A.E., Andersson S.E., Malmstrom P.U., Wester H.K. (2009). A novel strategy based on histological protein profiling in-silico for identifying potential biomarkers in urinary bladder cancer. BJU Int..

[36] Mezheyeuski A., Bergsland C.H., Backman M., Djureinovic D., Sjoblom T., Bruun J., Micke P. (2018). Multispectral imaging for quantitative and compartment-specific immune infiltrates reveals distinct immune profiles that classify lung cancer patients. J. Pathol..

