# Supplementary material for: High Density of NRF2 Expression in Malignant Cells Is Associated with Increased Risk of CNS Metastasis in Early-Stage NSCLC
Source: Cancers (Basel). 2021 Jun 24;13(13):3151. doi: 10.3390/cancers13133151 (PMC8268817; doi:10.3390/cancers13133151)

Supplementary Materials

Figure S1: Distribution of NRF2+/CK+ cell density in the whole-tissue core compartment.

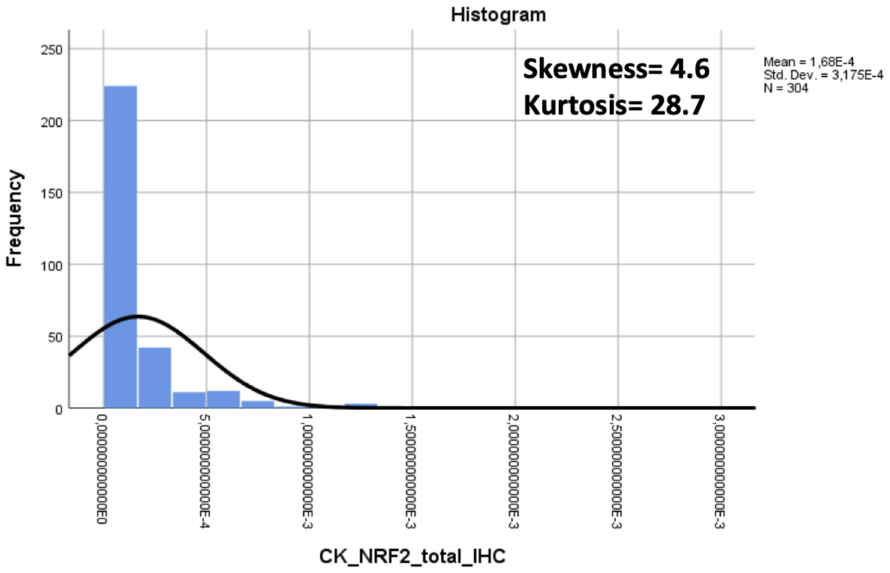

Figure S2: Receiver operating characteristic (ROC) curve demonstrating the sensitivity and specificity of the median NRF2+/CK+ cell density in the whole-tissue compartment as a cut-off value on the probability of developing brain metastases.

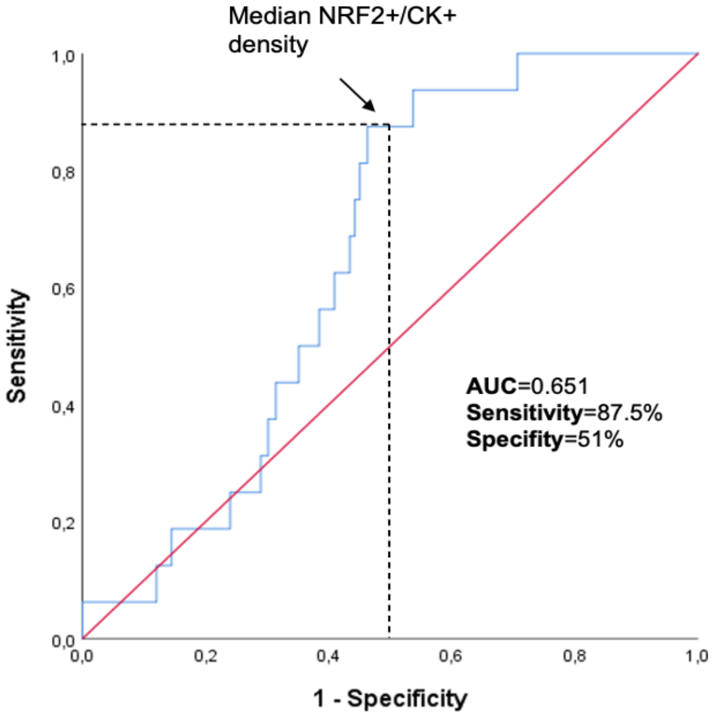

Supplement: Supplementary file 1 [file cancers-13-03151-s001.zip › cancers-1221610-supplementary.pdf]
